# M1/M2 macrophages and associated mechanisms in congenital bicuspid aortic valve stenosis

**DOI:** 10.3892/etm.2014.1529

**Published:** 2014-02-10

**Authors:** RUI WANG, WEN CHEN, ZHIFEI MA, LIANGPENG LI, XIN CHEN

**Affiliations:** Department of Thoracic and Cardiovascular Surgery, Nanjing Hospital Affiliated to Nanjing Medical University, Nanjing, Jiangsu 210006, P.R. China

**Keywords:** congenital bicuspid aortic valve, tricuspid aortic valve, macrophage, inflammation, neovascularization

## Abstract

The aim of this study was to observe macrophage infiltration in congenital bicuspid aortic valve (CBAV) stenosis. M1/M2 macrophage distribution, inflammatory cytokine expression and the role of M1 macrophages during CBAV stenosis were also explored. The experimental and control groups comprised 30 severely stenotic CBAVs and 30 severely stenotic tricuspid aortic valves (TAVs), respectively. Histological and morphological changes were assessed using hematoxylin-eosin (HE) staining and mRNA levels of vascular endothelial growth factor (VEGF) were examined using the quantitative polymerase chain reaction. Nonspecific, M1 and M2 macrophages were monitored using cluster of differentiation (CD)68, inducible nitric oxide synthase (iNOS) and CD163 staining, respectively. Endothelial nitric oxide synthase (eNOS), interleukin (IL)-10, arginase (Arg)-1 and macrophage colony-stimulating factor (M-CSF) were also examined using immunohistochemical staining. Of note, HE staining revealed a higher cell density and neovascularization was more common in CBAVs than TAVs. At the mRNA level, VEGF expression was two-fold higher in CBAVs relative to that in TAVs (P=0.02). Furthermore, CD68 and iNOS were significantly higher in CBAVs compared with TAVs (P=0.029 and 0.021, respectively), while CD163 expression was lower in CBAVs (P=0.033). In addition, eNOS expression was higher and Arg-1, IL-10 and M-CSF expression were lower in CBAVs compared with TAVs (all P<0.0001). The present study suggested that CBAVs are associated with a higher total and M1 macrophage density and a lower M2 macrophage density than TAVs, and that M1 macrophage infiltration may contribute to calcification of CBAVs.

## Introduction

Congenital bicuspid aortic valve (CBAV) is a common congenital heart malformation that occurs in ~2% of the population (1). Compared with normal tricuspid aortic valves (TAVs), CBAV is more likely to progress to valve stenosis, regurgitation or endocarditis (2). Stenosis is the most frequent complication of CBAV, often necessitating surgical intervention relatively early in life compared with TAVs (3). The early onset of severe stenosis reflects the more rapid progression of valve stenosis that is frequently observed in patients with CBAV. For this reason, CBAV has been a particular focus of study. Valve calcification and atherosclerosis have pathological similarities, including the destruction and apoptosis of endothelial cells, infiltration of inflammatory cells, new blood vessel formation, lipid plaque deposition and eventual calcification and ossification. Therefore, macrophages have an important role in the process of inflammatory factor infiltration (4–6).

Macrophages within the lesion are heterogeneous in nature, and are able to differentiate into either a pro-inflammatory (M1) subtype, also known as a classically activated subtype, or an anti-inflammatory alternatively activated subtype (M2) according to their microenvironment (7). Classically-activated pro-inflammatory macrophages may be induced by incubation with the Th1 cytokines interferon γ (IFN-γ) (for M1) and tumour necrosis factor (TNF-α) which work synergistically together and are both required to induce maximal macrophage activation. These macrophages are characterized by their secretion of pro-inflammatory cytokines, nitric oxide and high capacity to present antigen. By contrast, alternatively-activated anti-inflammatory macrophages may be induced with the Th2 cytokines interleukin 4 (IL-4) (for M2) and IL-13 and are characterized by their secretion of anti-inflammatory cytokines such as IL-10 and TGF-β (8). IL-4 stimulates the activity of the nuclear receptor peroxisome proliferator-activated receptor (PPARγ) which has been shown to mediate alternative macrophage activation as well as the transcriptional repression of several pro-inflammatory transcription factors. Therefore, macrophages may be categorized into the classically activated type (M1) and the alternatively activated type (M2). M1 macrophages promote inflammation and remove pathogenic microorganisms, while M2 macrophages inhibit inflammation and heal wounds (9,10). Numerous studies have investigated the distribution and pathogenic mechanisms of macrophage subtypes in atherosclerosis and cancer (11–13). The present study aimed to determine whether inflammation, defined by inflammatory cell infiltration and neovascularization, is increased in stenotic CBAV, and to explore the classification and possible mechanisms of differentiation of M1/M2 macrophages during the process of CBAV stenosis.

## Materials and methods

### Study population and data collection

From January 2011 to December 2012, a total of 30 patients with severely stenotic CBAVs underwent successful aortic valve replacement at Nanjing Hospital Affiliated to Nanjing Medical University (Nanjing, China). A group comprising 30 patients with severely stenotic TAVs was enrolled in parallel, and all CABVs and TAVs were included for further analyses. There were no statistically significant differences between the CABV and the TAV group in preoperative echocardiography, cardiovascular risk factors, other underlying diseases or the use of relevant cardiovascular medications (P>0.05; Table I). All cases were diagnosed using clinical and pathological evidence. This study conformed to the principles outlined in the Declaration of Helsinki. All of the retrospective review of the clinical data involving human samples were approved by the Human Research Ethics Committee of Nanjing Medical University, and written informed consent was obtained from each patient and the families of the prospective heart donors.

### Specimen collection and sectioning

All aortic valve specimens were fixed with 4% formaldehyde, followed by gradient alcohol dehydration, paraffin embedding and preparation of 3-μm sections.

### Hematoxylin-eosin (HE) staining

For HE staining, sections were deparaffinized, hydrated, stained with hematoxylin for 5 min and rinsed with water for 10 min. The sections were then decolorized using 1% HCl, rinsed with water for 10 min, stained with eosin for 1 min, dehydrated in gradient alcohols, and cleared in xylene prior to being sealed with neutral gum (Beijing Zhongshan Golden Bridge Biotechnology, Beijing, China).

### Immunohistochemical staining

Sections were deparaffinized, hydrated and rinsed twice in phosphate-buffered saline (PBS) for 5 min. The sections were then boiled to retrieve antigens and incubated with sheep serum to block nonspecific staining. Primary antibodies to cluster of differentiation (CD)68 (Beijing Zhongshan Golden Bridge Biotechnology), CD163 (Beijing Zhongshan Golden Bridge Biotechnology), inducible nitric oxide synthase (iNOS, 1:400; Beijing Zhongshan Golden Bridge Biotechnology), endothelial nitric oxide synthase (eNOS, 1:200; Santa Cruz Biotechnology, Inc., Santa Cruz, CA, USA), interleukin-10 (IL-10, 1:100; Santa Cruz Biotechnology, Inc.), arginase-1 (Arg-1, 1:2,000; Santa Cruz Biotechnology, Inc.) and macrophage colony-stimulating factor (M-CSF, 1:50; Santa Cruz Biotechnology, Inc.) were added. A diluted primary antibody was used as a reference and the sections were incubated at 4°C overnight.

The sections were subsequently brought to room temperature for 30 min and rinsed with PBS four times for 5 min, followed by the addition of anti-rabbit, -goat or -rat secondary antibodies (Santa Cruz Biotechnology, Inc.). The sections were incubated at room temperature for 15–60 min (according to the secondary antibody) and rinsed with PBS four times for 5 min. The sections were then stained with hematoxylin while being observed under a microscope, dehydrated, cleared and sealed.

Specimens were scored as positive when the cytoplasm and nucleus contained yellow or brown particles. Five samples were selected at random and viewed under high magnification (x400; Binocular Biological Microscope XSZ-PW150; Nikon, Tokyo, Japan). Positive cells were classified using the Fromowitz semi-quantitative method, which considers staining intensity and the percentage of positive cells (14). The staining intensity was scored as follows: No staining or similar to the background, 0 points; lightly stained or slightly higher than the background, 1 point; moderately stained or significantly higher than the background, 2 points; strongly or deeply stained, 3 points. The percentage of positive cells was scored as follows: <5%, 0 points; 5–25%, 1 point; 26–50%, 2 points; 51–75%, 3 points; >75%, 4 points. The scores for the two items were combined to derive a final score that was divided into four grades: 0–1 point, (−); 2–3 points, (+); 4–5 points, (++); 6–7 points, (+++).

### Real-time polymerase chain reaction (qPCR)

The TRIzol^®^ method was used to extract total RNA. Fluorescence qPCR was performed according to the instructions of the 2X One-Step qRT-PCR SYBR-Green kit (Tiangen Biotech (Beijing) Co., Ltd., Beijing, China), using primers designed by Shanghai Biological Engineering (Shanghai, China). Each reaction included 10 μl 2X One-Step SYBR-Green Master Mix, 300 ng template RNA, 0.4 μl upstream primer (10 μmol/l), 0.4 μl downstream primer (10 μmol/l) and 1 μl RT-PCR mix, and was adjusted to 20 μl with RNase-free water. RT-PCR was performed as follows: 42°C for 20 min; 95°C for 5 min; 40 cycles at 95°C for 15 sec, 55°C for 15 sec and 72°C for 20 sec; 95°C for 1 min, 55°C for 30 sec and 95°C for 30 sec. Each sample was evaluated in triplicate. The amplification and melting curves were plotted and the β-actin gene was used as an internal control. qPCR was performed using a 3.0×10^−4^ to 3.0×10^−5^ dilution series and five temperature gradients, the temperature of 55°C five additional cycles were carried out.

### Statistical analysis

All data are presented as the mean ± standard deviation (SD). For two-group comparisons, Gaussian samples were compared using the two-tailed t-test, while non-Gaussian samples were compared using the Mann-Whitney nonparametric test. Statistical analyses were performed using SPSS 17.0 software (SPSS, Inc., Chicago, IL, USA). P<0.05 was considered to denote a statistically significant difference.

## Results

### HE staining

**Figure 1:**
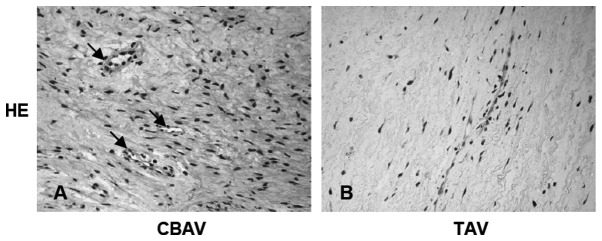
HE-stained aortic tissue specimens. The (A) CBAV specimen contains more infiltrating cells and a disordered cell arrangement, and the appearance of new capillaries (black arrows) is more typical than in the (B) TAV specimen. Magnification, ×400. HE, hematoxylin-eosin; CBAV, congenital bicuspid aortic valve; TAV, tricupsid aortic valve.

Histological morphology revealed calcification in all valves; however, more severe calcification was observed in CBAVs. HE staining revealed a higher cell density in CBAVs (Fig. 1A) than TAVs (Fig. 1B), and more neovascularization was exhibited in the CBAV (Fig. 1A) compared with the TAV group (Fig. 1B).

### VEGF expression

**Figure 2:**
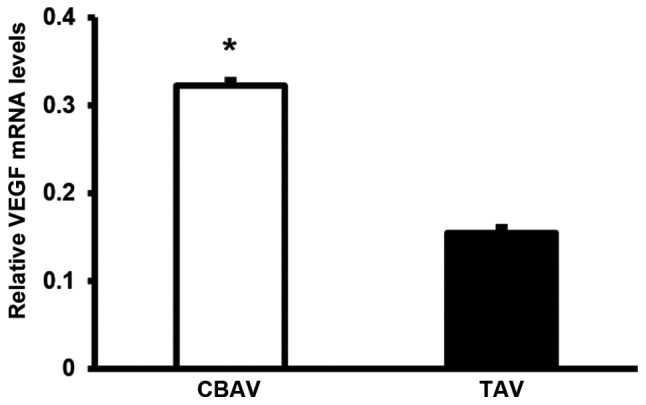
Upregulation of VEGF expression in CBAV. Quantitative polymerase chain reaction analysis of VEGF in the CABV and TAV groups (n=4/experimental group). Data are expressed as the mean ± standard deviation and represent the typical results of three to four different experiments. ^*^P<0.05 vs. TAV. VEGF, vascular endothelial growth factor; CBAV, congenital bicuspid aortic valve; TAV, tricupsid aortic valve.

Despite the severe calcification and formalin fixative, expression levels of VEGF were evaluable in 19 and 22 specimens in the TAV and CBAV groups, respectively. mRNA expression of VEGF in the CBAV specimens was two-fold that observed in the TAV specimens (P=0.02; Fig. 2). These results indicate that a positive correlation may exist between valve inflammation and neovascularization.

### Total and M1/M2 macrophage levels

**Figure 3:**
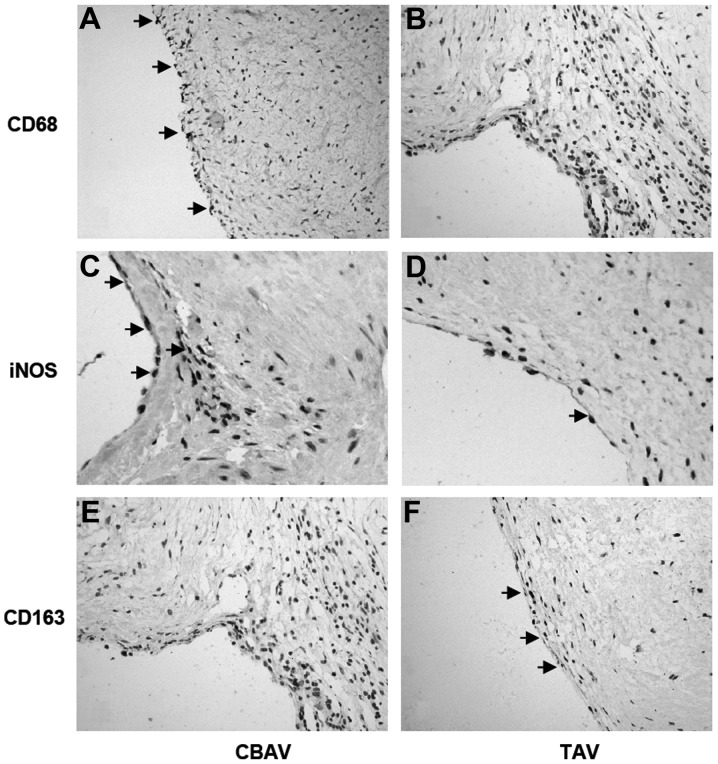
Immunohistochemical staining of CD68, iNOS and CD163 in aortic tissue. CD68 and iNOS expression was higher in the CBAV specimen (A and C, respectively) than in the TAV specimen (B and D, respectively). In addition, CD163 expression was lower in the (E) CBAV specimen than the (F) TAV specimen (black arrows indicate the expression of CD68/iNOS/CD163; magnification, ×400). iNOS, inducible nitric oxide synthase; CD, cluster of differentiation; CBAV, congenital bicuspid aortic valve; TAV, tricupsid aortic valve.

CD68, iNOS and CD163 staining was used to evaluate total, M1 and M2 macrophages, respectively. In the CBAV group, the density and distribution of macrophages (CD68) was significantly higher and wider than in the TAV group (4.53±1.92 versus 3.00±1.41, P=0.029; Fig. 3A and B), spanning the lining layer of the aortic valve. This was consistent with the results for the iNOS-labeled M1 macrophages in CBAVs compared with TAVs (3.20±2.27 versus 1.40±0.99, P=0.021; Fig. 3C and D). However, significantly fewer CD163-labeled M2 macrophages were observed in CBAVs than in TAVs (1.13±0.92 versus 2.27±1.58, P=0.033; Fig. 3E and F). Collectively, these data suggest that iNOS-labeled M1 macrophages may participate in CBAV stenosis.

### Expression of M-CSF and other inflammatory factors associated with macrophage transformation

**Figure 4:**
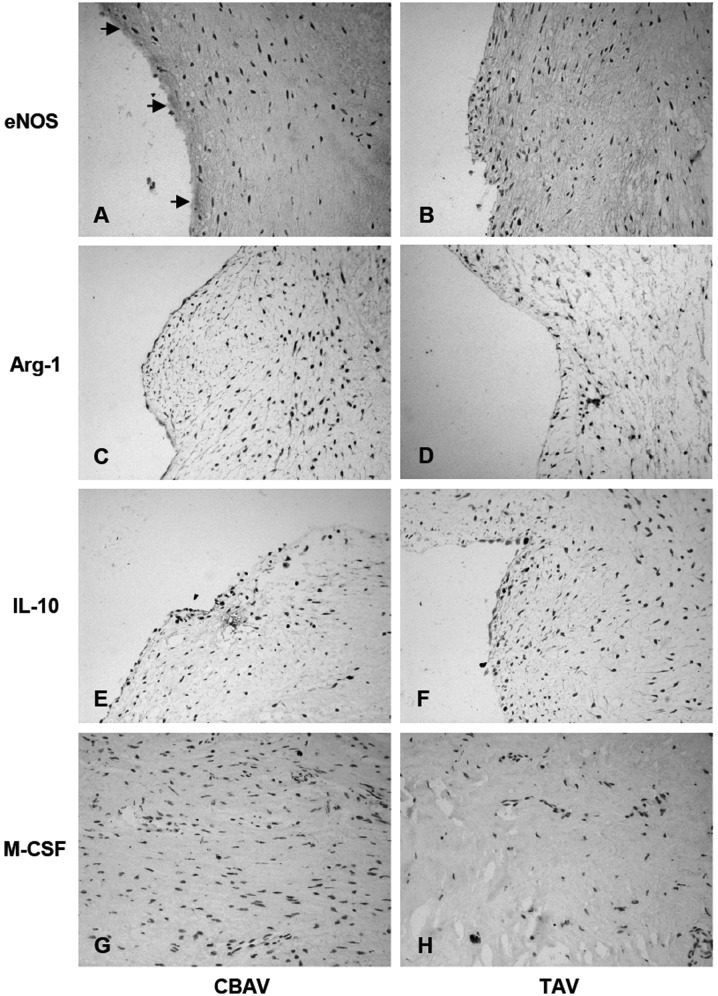
Immunohistochemical staining of eNOS, Arg-1, IL-10 and M-CSF in aortic tissue. eNOS expression was significantly higher in the (A) CBAV specimen than the (B) TAV specimen. Arg-1, IL-10 and M-CSF expression was slightly lower in the CBAV specimen (C, E and G, respectively) than the TAV specimen (D, F and H, respectively) (black arrows indicate the expression of eNOS/Arg-1/IL-10/M-CSF; magnification, ×400). eNOS, endothelial nitric oxide synthase; Arg-1, arginase-1; IL-10, interleukin-10; M-CSF, macrophage colony-stimulating factor; CBAV, congenital bicuspid aortic valve; TAV, tricupsid aortic valve.

In CBAVs, eNOS expression was significantly higher than in TAVs (4.93±2.25 versus 2.00±1.41, P=0.0003; Fig. 4A and B). However, Arg-1 expression was slightly lower in CBAVs than TAVs (1.40±1.06 versus 3.53±1.51, P=0.0009; Fig. 4C and D), as evidenced by the expression of IL-10 (2.07±0.88 versus 4.67±1.54, P=0.0007; Fig. 4E and F) and M-CSF (2.40±1.06 versus 4.47±1.23, P=0.0006; Fig. 4G and H). Thus, inflammatory responses in CBAV stenosis may result from the generation of other inflammatory factors and inhibition of M1/M2 macrophage transformation.

## Discussion

CBAV is a common congenital heart malformation. Individuals with CBAV are more susceptible to aortic valve disease at a young age, under certain circumstances even progressing to aortic aneurysm and aortic dissection. Over the past decades, studies have demonstrated that aortic valve calcification and atherosclerosis are characterized by similar pathological processes, with the two conditions being initiated by disruption of the basement membrane and lipid deposition, and mediated by inflammatory cell infiltration and neovascularization (15–17). Experimental and clinical studies have also shown that the structure and geometric shape of CBAVs differ from that of normal TAVs (1,18,19). CBAV leaflets experience higher shear stress, which leads to inflammatory cell infiltration and the damage and apoptosis of endothelial cells (20). In addition, CBAV calcification has been associated with VEGF gene expression (21).

Macrophages are one of the most important components of the innate immune system. The M1 and M2 macrophage subtypes participate in the regulation of numerous biological processes. M1 macrophages are involved in inflammation and bacterial clearance, and are stimulated by lipopolysaccharide and/or interferon γ, which stimulates iNOS expression. M2 macrophages are stimulated by IL-4 and IL-13, and inhibit the inflammatory reaction and promote the repair of damaged tissue (22,23). However, little is known about the functions of M1/M2 macrophages during the pathogenesis of CBAV stenosis.

The present study indicates that CD68 expression was higher in CBAVs than in TAVs, as demonstrated by iNOS expression. However, CD163 expression was lower in CBAVs than TAVs. These results suggest that M1 macrophage infiltration may contribute to calcification of CBAVs. Of note, CBAVs develop earlier and more easily than TAVs, and exhibit more severe calcification. This is in concurrence with the hypothesis that inflammatory cell infiltration is one of the most important aspects of calcification. Numerous studies have demonstrated that M1 macrophages are mainly involved in the inflammatory response, whereas M2 macrophages participate in the inhibition of the inflammatory response (22,23). Therefore, it may be suggested that M1 macrophages actively participate in promoting inflammation in CBAV, thus aggravating valvular lesions.

With regard to the function of macrophages during the inflammatory process, with the exception of direct phagocytosis, macrophages may participate and modify reactions through cytokine secretion and regulation. In addition, different macrophage subtypes may secrete and regulate different inflammatory factors; for example, M1 macrophages express high levels of iNOS and secrete the inflammatory cytokines tumor necrosis factor-α, IL-1, IL-6 and IL-12, whereas M2 macrophages express high levels of IL-10 and tumor growth factor-β (22). In the present study, eNOS expression in CBAVs was high, while that of IL-10 was relatively low. M1 macrophages perpetuate the inflammatory response by upregulating eNOS expression and inhibiting IL 10 expression. The relatively low expression of IL-10 in CBAVs could not effectively inhibit cytokine production by activated macrophages, leading to the perpetuation of the inflammatory response. By contrast, the low expression of IL-10 in TAVs may led to the suppression of the inflammatory response.

Studies *in vivo* have demonstrated that iNOS and Arg-1, expressed by M1 and M2 macrophages, respectively, competitively bind with L-arginine and exert pro- and anti-inflammatory effects, respectively, through their metabolites (24). iNOS degrades L-arginine into NO and reactive oxygen species, which may promote inflammation and the removal of invading microorganisms. However, Arg-1 degrades L-arginine into L-ornithine, which may promote collagen synthesis and the restoration of damage. In the present study, Arg-1 expression was low in CBAVs but slightly higher in TAVs. From these results, it may be concluded that Arg-1 inhibits the inflammatory response in TAVs. Conversely, the dominant M1 macrophages in CBAVs may lead to a long-term valve inflammatory formation, involving competitive combine with L-arginine degradation and Arg-1 suppression, respectively.

Macrophages exhibit plasticity, and studies have shown that peroxisome proliferators are able to downregulate the proportion of M1 to M2 macrophages, promote differentiation of M1 macrophages into M2 macrophages (25), exert an anti-inflammatory effect and weaken the inflammatory responses of M1 macrophages (26). Randolph *et al* (27) demonstrated that CD163 expression was enhanced following stimulation with M-CSF, IL-10 and dexamethasone; therefore, M-CSF may stimulate transformation of mature macrophages into M2 macrophages. In the present study, M-CSF expression was significantly lower in CBAVs than in TAVs, which indicates that, during CBAV calcification, M-CSF may decrease the transformation of M1 macrophages to M2 macrophages and maintain the valvular injury caused by M1 macrophages.

Notably, stenotic CBAVs were found to be associated with increased neovascularization when compared with TAVs. Consistent with this observation, another histopathological study revealed an association between neovascularization and inflammatory changes during the valvular calcification process, and the formation of new blood vessels occurred significantly faster in stenotic valves than in normal valves (28). Thus, these data indicate that a positive correlation exists between valve inflammation and neovascularization. Endothelial injury is involved in the initiation of valve stenosis. The VEGF family has a vital role in physiological and pathological conditions, due to the ability to promote endothelial cell migration and proliferation, and increase vascular permeability and thrombosis. Macrophages secrete a variety of matrix proteins that activate VEGF and trigger the osteoblastic transdifferentiation of vascular smooth muscle cells. This partially explains the VEGF data from the present study, with inflammatory cells and neovessels in close proximity to valve calcification, and suggests that valve inflammation is responsible for subsequent calcification and stenosis.

In conclusion, the present study demonstrates that severe aortic stenosis in CBAVs is associated with a more aggressive inflammatory process that involves greater inflammatory cell infiltration and neovascularization when compared with TAVs. Of note, the results show the importance of M1/M2 macrophage distribution, inflammatory cytokine expression and the role of M1 macrophages in the development of CBAV stenosis. These results may help further elucidate the more frequent onset and rapid progression of atherosclerosis and severe aortic valve calcification observed in patients with CBAV.

## Figures and Tables

**Table I:** Clinical and demographic characteristics of the patients.

	CBAV (n=30)	TAV (n=30)	P-value
Age (years)	61±9	59±11	0.44
Female (n)	13	11	0.60
Risk factors			
Hypertension (n)	11	9	0.58
Hypercholesterolemia (n)	10	10	1.00
Diabetes mellitus (n)	4	6	0.49
Chronic kidney disease (n)	1	0	0.31
Smoking (n)	11	14	0.43
Medical therapy			
Diuresis (n)	27	29	0.30
ARB (n)	4	5	0.72
ACEI (n)	6	5	0.74
UCG parameters			
LVEF (%)	52±6	54±4	0.13
AVA (cm)	0.88±0.2	0.81±0.3	0.29
Mean gradient (mmHg)	55±16	59±14	0.31
Aortic annulus diameter (cm)	23±2.3	24±1.9	0.07

CBAV, congenital bicuspid aortic valve; TAV, tricupsid aortic valve; ARB, angiotensin receptor blocker; ACEI, angiotensin-converting enzyme inhibitor; UCG, ultrasound cardiography; LVEF, left ventricular ejection fraction; AVA, aortic valve area.
